# Cleavage of Occludin by Cigarette Smoke-Elicited Cathepsin S Increases Permeability of Lung Epithelial Cells

**DOI:** 10.3390/antiox12010005

**Published:** 2022-12-21

**Authors:** Paul Bigot, Simon Chesseron, Ahlame Saidi, Damien Sizaret, Christelle Parent, Agnès Petit-Courty, Yves Courty, Fabien Lecaille, Gilles Lalmanach

**Affiliations:** 1 Faculty of Medicine, University of Tours, 37000 Tours, France; 2 Team “Proteolytic Mechanisms in Inflammation”, INSERM, UMR1100, Research Center for Respiratory Diseases (CEPR), 37000 Tours, France; 3 Pathological Anatomy and Cytology, The University Hospital Center of Tours, 37000 Tours, France; 4 Team “Aerosol therapy and Biotherapeutics for Respiratory Diseases”, INSERM, UMR1100, Research Center for Respiratory Diseases (CEPR), 37000 Tours, France

**Keywords:** cathepsin, chronic obstructive pulmonary disease (COPD), cigarette smoke, emphysema, epithelial permeability, lung, occludin, protease

## Abstract

Background. Chronic obstructive pulmonary disease (COPD) is an irreversible disease mainly caused by smoking. COPD is characterized by emphysema and chronic bronchitis associated with enhanced epithelial permeability. Hypothesis. Lung biopsies from smokers revealed a decreased expression level of occludin, which is a protein involved in the cohesion of epithelial tight junctions. Moreover, the occludin level correlated negatively with smoking history (pack-years), COPD grades, and cathepsin S (CatS) activity. Thus, we examined whether CatS could participate in the modulation of the integrity of human lung epithelial barriers. Methods and results. Cigarette smoke extract (CSE) triggered the upregulation of CatS by THP-1 macrophages through the mTOR/TFEB signaling pathway. In a co-culture model, following the exposure of macrophages to CSE, an enhanced level of permeability of lung epithelial (16HBE and NHBE) cells towards FITC-Dextran was observed, which was associated with a decrease in occludin level. Similar results were obtained using 16HBE and NHBE cells cultured at the air–liquid interface. The treatment of THP-1 macrophages by CatS siRNAs or by a pharmacological inhibitor restored the barrier function of epithelial cells, suggesting that cigarette smoke-elicited CatS induced an alteration of epithelial integrity via the proteolytic injury of occludin. Conclusions. Alongside its noteworthy resistance to oxidative stress induced by cigarette smoke oxidants and its deleterious elastin-degrading potency, CatS may also have a detrimental effect on the barrier function of epithelial cells through the cleavage of occludin. The obtained data emphasize the emerging role of CatS in smoking-related lung diseases and strengthen the relevance of targeting CatS in the treatment of emphysema and COPD.

## 1. Introduction

Besides exposure to dust, industrial, or domestic particles, chronic obstructive pulmonary disease (COPD) is primarily attributable to the inhalation of chemicals and oxidants found in cigarette smoke (CS) [1]. COPD numbers among the foremost causes of death and morbidity worldwide and no therapies are currently available [2,3]. COPD is characterized by a progressive and irreversible obstruction of the airways (chronic bronchitis), a destruction of the alveolar walls (emphysema), and mucus hyper secretion. This progressive and irreversible airflow blockage is combined with exacerbation (bacterial or viral infections) episodes [4]. The mechanistic basis underlying COPD is complex and involves oxidative stress (oxidant/antioxidant imbalance) and protease/antiprotease imbalance, but also environmental, genetic, and epigenetic factors [5,6,7].

Cysteine cathepsins belong to a family consisting of 11 members (clan CA, family C1) [8]. Their catalytic mechanism requires the presence of a catalytic dyad composed of a nucleophilic Cys25 and His159 (papain numbering), which is assisted by Asn175. Cysteine cathepsins are primarily lysosomal proteases. Nevertheless, they can be secreted and found in the pericellular environment as soluble enzymes or associated with cell surfaces [9,10]. Besides their conventional recycling tasks, cysteine cathepsins are involved in specialized biological roles including the maturation of some prohormones, apoptosis, and antigen presentation [11,12]. They also participate in a wide range of diseases, such as rheumatoid arthritis, osteoporosis, or myocardial infarction [13,14,15]. Accordingly, some cysteine cathepsins are considered to be valuable biomarkers and/or therapeutic targets [16,17,18,19,20]. Along with regulation by specific protease inhibitors of the cystatin family [21,22,23,24], their activity can be governed by glycosaminoglycans (GAGs) and the redox imbalance associated with oxidative stress [25,26,27]. Cysteine cathepsins are expressed in numerous pulmonary tissues, and their importance in lung homeostasis and diseases has emerged over the past two decades (for review: [28,29]). Their dysregulation contributes to lung illnesses (e.g., emphysema, chronic bronchitis, idiopathic pulmonary fibrosis, cystic fibrosis, and asthma), as they participate in the degradation of antimicrobial peptides and proteins as well extracellular matrix and basement membrane components [30,31,32,33,34,35]. Likewise, the deleterious effects of CatS (EC 3.4.22.27), which is a potent elastase involved in alveolar remodeling, have been reported in various lung diseases [33]. Overexpression of CatS was observed in murine experimental emphysema, while its inhibition reduced the severity of emphysema and inflammation [34,35]. Recently, we demonstrated that CatS expression, as well its enzymatic activity, is amplified in both non-COPD and COPD smokers compared to never-smokers, and correlates positively with smoking history [36]. Despite the presence of a reactive nucleophilic cysteine (Cys25) within its active site, the elastinolytic activity of CatS is partially preserved after exposure to cigarette smoke or major oxidants of cigarette smoke and subsequently may contribute to parenchymal destruction during emphysema [26,36,37]. Thus, the pharmacological inhibition of CatS could be a valuable therapeutic option with which to reduce the severity of emphysema [33,38,39,40].

In previous studies, it was reported that CatS participates in the degradation of E-cadherin—proven via a mouse model of pancreatic cancer [41]—and may mediate blood–brain barrier transmigration through the proteolytic processing of JAM-B-favoring brain metastasis [42]. On the other hand, besides the recruitment of cells that contribute to chronic airway inflammatory response and aberrant lung tissue remodeling, CS increases epithelial permeability during emphysema [43]. Hence, in accordance with this line of research, it is prudent to scrutinize the signaling pathways leading to lung CatS upregulation in human macrophages exposed to cigarette smoke extract (CSE) and to examine whether CatS could participate in the modulation of the integrity of epithelial barriers in COPD, despite an unfavorable oxidizing environment. Therefore, the aim of this work was to identify a CatS target within junction proteins and to assess the potential impact of CatS on the permeability of lung epithelial cells.

## 2. Materials and Methods

Details of the materials and methods employed are provided in the Supplemental Material online (see references [17,36,44,45,46,47,48,49,50,51,52,53]).

### 2.1. Ethical Statement

Tumor-free bronchial biopsies were obtained from patients with lung tumors with or without COPD and who underwent surgery for non-small cell lung cancer in the Thoracic Surgery Department (The University Hospital Center, Tours, France) [54]. The study was conducted with the agreement of the local bioethical committee of the University Hospital Center (Tours, France) and tissue collections were declared to the French Ministry of Higher Education, Research, and Innovation (approval DC-2008-308). Written informed consent was obtained from each patient in compliance with the Declaration of Helsinki. Ethical standards set out in the Declaration of Helsinki were respected. Data on age, histological examinations, smoking, and COPD status were recorded for each patient.

### 2.2. Clinical Status

Smoking history was defined as pack-years (number of packs of cigarettes smoked per day multiplied by the number of years the person has smoked). Lung capacity was established by a spirometry test by calculating FEV_1_ (forced expiratory volume in one second) vs. FVC (forced vital capacity) (FEV_1_/FVC ratio of <0.7 for COPD patient). The GOLD (Global Initiative for Chronic Obstructive Lung Disease) system categorizes airflow limitation into stages: GOLD I is mild with an FEV_1_ (forced expiratory volume in one second, % predicted) ≥ 80%, GOLD II is moderate (50% ≤ FEV_1_ < 80% predicted) and Gold III is severe (30% ≤ FEV_1_ < 50% predicted). The cohort was diagnosed as consisting of 12 non-COPD patients (6 smokers and 6 non-smokers) and 16 COPD patients (6 GOLD stage I, 5 GOLD stage II, and 5 GOLD stage III). All COPD patients were active smokers.

### 2.3. Handling of Clinical Specimens

Lung biopsies corresponding to non-tumor tissues from patients who underwent surgery for non-small cell lung cancer were collected at least 3 cm away from the tumor. Twenty-eight tissue samples, which corresponded to a previously analyzed subset [36], were reviewed by the Department of Pathological Anatomy and Cytology (The University Hospital Center, Tours, France) to assess the absence of tumor cells. Samples were frozen in liquid nitrogen and stored at −80 °C. Tissues were further embedded in Tissue-Tek OCT (Sakura Finetek Europe, Alphen aan den Rijn, The Netherlands); then, 4 µm sections were prepared (Leica CM3050 S Cryostat, Leica Bio, Nanterre, France). Tissue sections were incubated in a lysis buffer (RIPA buffer, Thermo Fisher systems France, Nanterre, France) and a protease inhibitor cocktail (Sigma-Aldrich) to preserve samples from uncontrolled degradation. Proteins were extracted from tissue samples by three freezing–thawing cycles (liquid nitrogen/37 °C water bath) and sonication on ice (VibraCell sonicator, Thermo Fisher scientific) before centrifugation (21,000× *g*, 4 °C, 20 min). Supernatants were collected and proteins were quantified by bicinchoninic acid assay (BCA) (Interchim, Montluçon, France) before immunoblot analysis. Alternatively, sections were incubated in PBS containing protease inhibitors (0.5 mM pefabloc, 0.5 mM EDTA, 0.04 mM pepstatin A, and 1 mM S-methyl thiomethanesulfonate). Cell-free soluble fractions were retrieved by centrifugation (21,000× *g*, 4 °C, 20 min). Proteins were quantified by BCA assay before measurement of CatS activity.

### 2.4. Cigarette Smoke Extract (CSE)

CSE was prepared and used extemporaneously [37]. Succinctly, mainstream smoke of three filter-research-grade cigarettes (reference: 3R4F; Kentucky Tobacco Research and Development Center at the University of Kentucky, Lexington, KY, USA) was bubbled in PBS (50 mL) in a sealed environment (average burning time/cigarette: ~8 min) and then sterile-filtered. The CSE solution was calibrated by measuring the absorbance of polycyclic compounds (mostly quinic acid and nicotine) (λ = 320 nm; Cary 100 spectrophotometer, Agilent Technologies, Les Ulis, France) and was then diluted in culture media to obtain the desired concentration [36].

### 2.5. Cells

Human THP-1 monocytic cell line (ATCC, Manassas, VA, USA) was provided by LGC Promochem (Molsheim, France). Normal human bronchial/tracheal epithelial (NHBE) cells were supplied by Lonza (Basel, Switzerland). The 16HBE cells employed correspond to a human bronchial epithelial cell line originally obtained from a 1-year-old male and immortalized with SV40 plasmid. The 16HBE cells were generously provided by Prof. Dieter C. Gruenert (University of California, San Francisco, CA, USA).

### 2.6. Statistical Analysis

Data were expressed as median ± quartile. The non-parametric Mann–Whitney U test was used for comparison between two groups, while comparisons between three or more groups were performed with Kruskal–Wallis test using GraphPad Prism 6.0 (GraphPad software, San Diego, CA, USA). Statistically significant correlation between two variables was determined with the non-parametric Spearman test. Differences were considered significant at *p*-values < 0.05, <0.01, and <0.001, and were represented as *, **, and ***, respectively.

## 3. Results

### 3.1. Clinical Characteristics of Patients

Tumor-free lung tissue samples were checked, and the absence of carcinoma was histologically reviewed for validation (Department of Pathologic Anatomy and Cytology, The University Hospital Center, Tours, France). Alternatively, diagnosis of COPD was made based on smoking history and forced expiratory volume in 1 s versus a forced vital capacity (FEV_1_/FVC) ratio of <0.7 determined via spirometry tests. The clinical characteristics of patients that were enrolled in the present study are summarized in Table 1. The patients of the cohort were divided, based on their smoking status, into twelve non-COPD patients (six non-COPD (S) smokers and six non-COPD (NS) never-smokers) and sixteen COPD patients (six GOLD stage I, five GOLD stage II, and five GOLD stage III patients). All sixteen COPD patients were smokers (current or former smokers). The number of pack-years (i.e., the number of packs of cigarettes smoked per day multiplied by the number of years the person has smoked) was not statistically distinctive in all the groups of smokers (including CS and former smokers (FS) with or without COPD), while FEV1 in the GOLD stage II and III patients was significantly decreased compared to the non-COPD patients and GOLD stage I patients. In addition, no significant differences in mean age were found between the groups of the cohort.

### 3.2. Occludin Expression Is Decreased in Lung Tissue of Smokers

**Figure 1:**
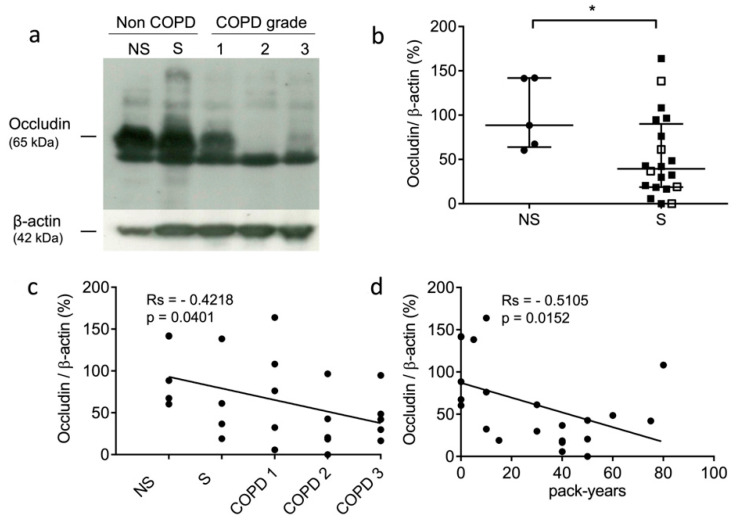
Expression level of occludin in lung tissue samples. (**a**): Western blot analysis (lung tissue lysates, 100 µg of total proteins/sample). Representative samples correspond to non-COPD never-smokers (NS), non-COPD smokers (S), and COPD patients (all being smokers with different stages of COPD severity): GOLD (Global initiative for chronic Obstructive Lung Disease) 1 (mild stage), GOLD 2 (moderate stage), and GOLD 3 (severe stage). (**b**): Densitometric analysis of occludin expression level (normalized data using β-actin as control; ImageJ software). Bars represent median ± quartile. Statistical significance was assessed using Mann–Whitney test (* *p* < 0.05). (**c**): Correlation between occludin level and patient status (NS, S, and COPD grades; GOLD 1–GOLD 3). (**d**): Correlation between occludin level and pack years (i.e., smoking history). Correlations were determined by linear regression and indicated by Spearman coefficient (Rs) and levels of significance (*p*).

The occludin levels of the non-COPD never-smokers, non-COPD smokers, and COPD smokers were analyzed by Western blot analysis. A higher amount of occludin was found for the non-COPD patients compared to the COPD patients (see representative samples: Figure 1a). the present results corroborated an immunohistochemistry (IHC) study that assessed that the expression level of occludin was significantly decreased in COPD patients [55]. Next, we compared the expression levels of occludin in the lung tissue samples of the never-smokers and smokers. The levels of occludin were significantly decreased in the lung tissue from smokers (*p* = 0.0411) (Figure 1b). Further, we evaluated whether the expression of immunoreactive occludin might be correlated with the GOLD stages and the smoking history of the patients. Occludin levels correlated negatively with COPD status and severity (rs = −0.4218, *p* = 0.0401) (Figure 1c). Similarly, occludin levels correlated negatively with pack-years of cigarette smoking (rs = −0.5105, *p* = 0.0152) (Figure 1d). Moreover, we carried out similar investigations on two tight junction proteins, claudin-1 and claudin-3, which participate in epithelial integrity and polarity via cohesive interactions between cells [56]. We did not find correlations between claudin-1 expression and smoking status (never-smokers vs. smokers) (*p* = 0.32), COPD severity (GOLD stages) (rs = −0.2977, *p* = 0.167), and pack-years (rs = −0.3349, *p* = 0.128) (see supplementary file: Figure S1). It was noticed that claudin-1 protein accumulation was previously reported for mice with defective cathepsin L activity (furless mice), suggesting that claudins might be proteolytic targets of cysteine cathepsins [57]. Likewise, no correlations were found between claudin-3 expression and smoking status (*p* = 0.5422), COPD severity (rs = −0.1605, *p* = 0.4336), and cumulative cigarette exposure (i.e., pack-years) (rs = −0.1624, *p* = 0.4379) (Figure S2).

### 3.3. The Occludin Protein Level Correlated Negatively with Cathepsin S Activity

**Figure 2:**
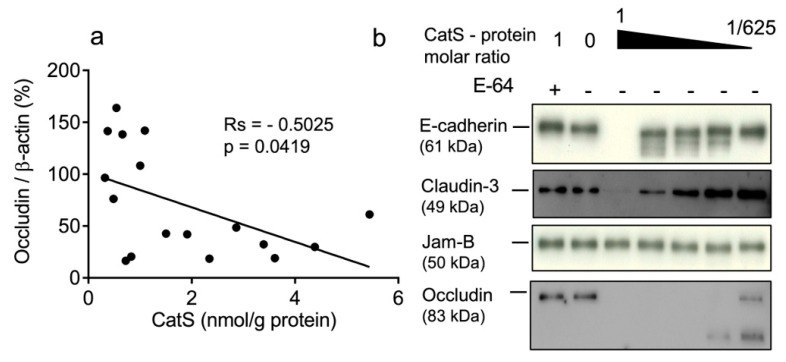
CatS activity negatively correlated with immunoreactive occludin level. (**a**): Correlation between CatS activity and occludin level in lung tissue lysates. Correlation was determined by linear regression and indicated by Spearman coefficient (Rs) and levels of significance (*p*). (**b**): in vitro hydrolysis of human recombinant E-cadherin, claudin-3, JAM-B, and occludin by CatS. Constant amount of junction proteins was incubated with CatS (molar ratio: 1:625 to 1:1) and incubated for 1 h at 37 °C in cell culture medium before their hydrolysis products were revealed by immunoblotting.

In a previous study, we demonstrated that CatS activity was significantly higher in the lung tissue of smokers (both non-COPD and COPD smokers) versus never-smokers and correlated positively with smoking history [36]. Therefore, we compared the protein levels of occludin and claudins-1 and -3 with CatS activity. No link was observed between the concentration of active CatS activity and claudin-1 expression (r_s_ = −0.03186, *p* = 0.906) (Figure S1) or between CatS activity and claudin-3 expression (r_s_ = −0.3475, *p* = 0.1333) (Figure S2). Contrary to claudins-1 and -3, the occludin levels in lung tissue were significantly and negatively associated with CatS activity (Figure 2a). Interestingly, our data corroborate a recent report suggesting that CatS inhibition restores the turnover of tight junction proteins including occludin of human glioblastoma cell lines [58]. Accordingly, we further investigated whether the occludin of epithelial tight junctions could be a proteolytic target of CatS. A pilot experiment was conducted before the analysis of lung epithelial cells. Assays were conducted at neutral pH in the culture medium to assess whether CatS could degrade recombinant occludin as well as archetypal adhesion/junction proteins, including claudin-3; E-cadherin, which was identified as a CatS substrate in a mouse model of pancreatic cancer [41]; and JAM-B, which was proposed as a CatS target promoting blood–brain barrier transmigration during brain metastasis [42]. As anticipated, CatS extensively hydrolyzed occludin in a dose-dependent manner, which is in agreement with our previous observation (Figure 2b). Conversely, CatS failed to degrade JAM-B in the culture medium at a neutral pH, even at a 1:1 stoichiometric CatS/JAM-B ratio, while E-cadherin was faintly cleaved by CatS in vitro. Of note, claudin-3 was only slightly hydrolyzed by CatS, supporting the lack of significant correlation between CatS activity and the claudin-3 protein level in the lung biopsies.

### 3.4. CSE Increased CatS Expression through the mTOR/TFEB Signaling Pathway

**Figure 3:**
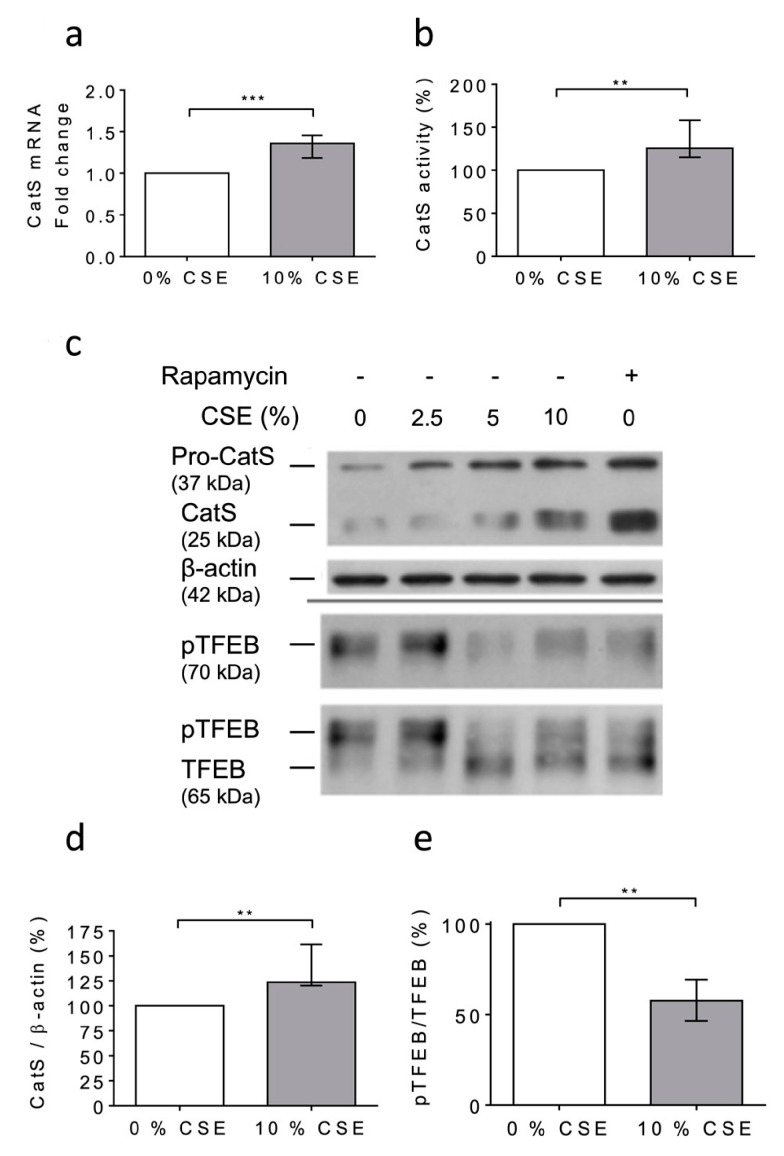
Cigarette smoke extract (CSE) triggered the expression and secretion of active CatS in THP1 cells through the mTOR/TFEB signaling pathway. (**a**): At day 6 after differentiation of THP1 monocytes into macrophages, CSE (10%) was added for 12 h. CatS transcription was analyzed by RT-qPCR. Data are expressed as relative fold change compared to the untreated control. (**b**): Peptidase activity of secreted CatS was measured using Z-Leu-Arg-AMC (20 µM) as substrate. (**c**): Macrophages were treated or untreated by CSE (10%) or rapamycin (50 nM) during 12 h. Protein levels (cell lysates) of CatS, β-actin, pTFEB (Ser 211), and TFEB were analyzed by Western blot analysis (β-actin, load control). A representative sample of five independent experiments is shown. (**d**): Densitometric analysis of the level of immunoreactive CatS (control: 0% CSE). (**e**): Densitometric analysis of the pTFEB/TFEB ratio (control: 0% CSE). For Figure 3d,e, normalized data correspond to the average of five independent WB experiments. Bars represent median ± quartile. Statistical significance was assessed using Mann–Whitney test (** *p* < 0.01; *** *p* < 0.001).

At day 6 after the PMA-dependent differentiation of human THP-1 monocytes [48,53], the macrophages were exposed to 10% CSE for 12 h. First, any dose-dependent decrease in the cell viability was observed (MTT assay); consequently, it was suggested that CSE did not exhibit lethal cellular effects under the present experimental conditions (Figure S3). Following the exposure of THP-1 cells to CSE, a significant increase in CatS mRNA level was observed (Figure 3a), which corroborated with enhanced peptidase CatS activity in the conditioned medium (*p* = 0.0079), thereby establishing that CSE elicited an increase in CatS secretion (Figure 3b). In addition, the Western blot analysis of the cell lysates showed a CSE-dependent upregulation of CatS protein level (both its ~25 kDa mature form and its ~37 kDa proform) (Figure 3c), which was confirmed by densitometry analysis (Figure 3d). According to previous reports, it was proposed that CSE could drive CatS secretion via the activation of P2X7 receptors [36,59]. On the other hand, it was recently established that nicotine, a major component of cigarette smoke, may upregulate CatS expression in vascular smooth muscle cells and in atherosclerotic plaques [52] via the inhibition of mTORC1 (mammalian target of rapamycin complex 1), which is associated with a decreased phosphorylation of TFEB (transcription factor EB), which, in turn, is primarily known as a central regulator of autophagy/lysosomal biogenesis [60]. THP-1 cells were treated with 10% CSE in the presence or absence of AZ 11645373, a P2X7 receptor antagonist. Immunoblot analysis showed that AZ 11645373 did not reduce the expression level of both CatS and pro-CatS, implying that the CSE-induced upregulation of macrophage CatS is not mediated by the MAPK signaling pathway through P2X7 receptor activation (data not shown). Alternatively, THP-1 cells were treated with CSE or with rapamycin (50 nM), an mTORC1 inhibitor of clinical relevance. Both CSE and rapamycin induced an increase in immunoreactive, mature CatS and its proform (Figure 3c). Conversely, a dose-dependent decrease in immunoreactive phosphorylated TFEB (pTFEB, ~70 kDa), corresponding to an impaired phosphorylation of TFEB (~65 kDa), was observed for the treated cells (Figure 3e), which is consistent with a previous report that indicated that the phosphorylation of TFEB at Ser211 by mTORC1 inhibits TFEB activity, while the pharmacological inhibition of mTORC1 activates TFEB by promoting its dephosphorylation [61]. Thus, the data show that the CSE-induced upregulation of macrophage CatS occurs through the mTORC1 signaling pathway.

**Figure 4:**
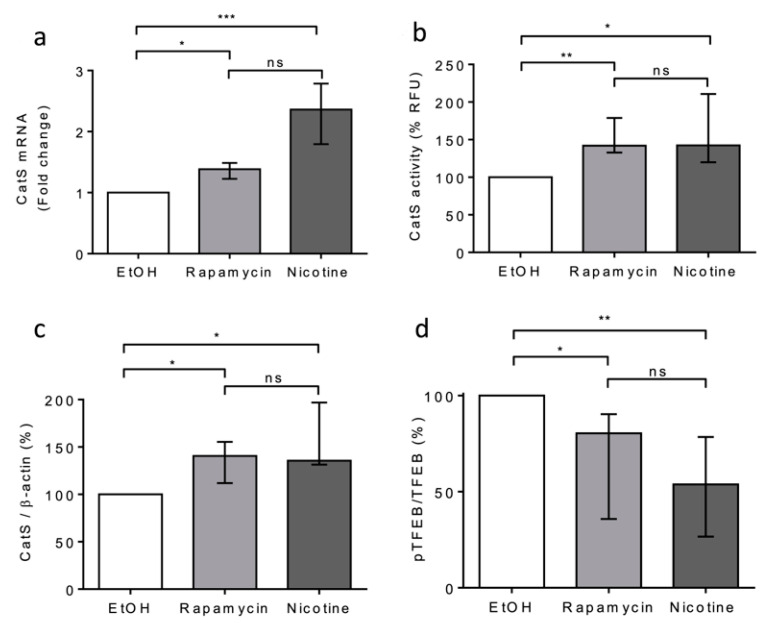
Nicotine-elicited upregulation and secretion of active CatS through the mTOR/TFEB signaling pathway in THP-1 macrophages. (**a**): THP-1 macrophages were treated with nicotine (5 µM) or rapamycin (50 nM) during 12 h and then lysed. CatS transcription was analyzed by RT-qPCR. Data are expressed as relative percentage compared to the untreated control (EtOH). (**b**): Enzymatic activity of secreted CatS was measured in supernatants of THP-1 cells. (**c**): Densitometric analysis of the mature CatS. Normalized data correspond to the average of five independent Western blot experiments (β-actin, load control). (**d**): Densitometric analysis of the pTFEB/TFEB ratio. Normalized data correspond to the average of five independent Western blot experiments. Bars represent median ± quartile. Statistical significance was assessed using Kruskal–Wallis test (* *p* < 0.05; ** *p* < 0.01; *** *p* < 0.001). ns: not significant.

Next, we compared the effects of rapamycin and nicotine on THP-1 cells. Both rapamycin and nicotine led to a significant increase in CatS mRNA expression, peptidase activity of secreted CatS, and its intracellular protein level (Figure 4a–c), which was associated with a decrease in TFEB phosphorylation (see pTFEB/TFEB ratio: Figure 4d). Taken together, our data indicate that CSE increased CatS expression through the mTOR/TFEB signaling pathway in THP-1 cells and suggest that the CSE-induced upregulation of CatS was partly elicited by nicotine, one of its main constituents, as observed for vascular smooth muscle cells [52].

### 3.5. Exposure of Human Lung Epithelial Cells to CatS Induced Both a Decrease in the Protein Level of Occludin and an Increase in Epithelial Permeability

**Figure 5:**
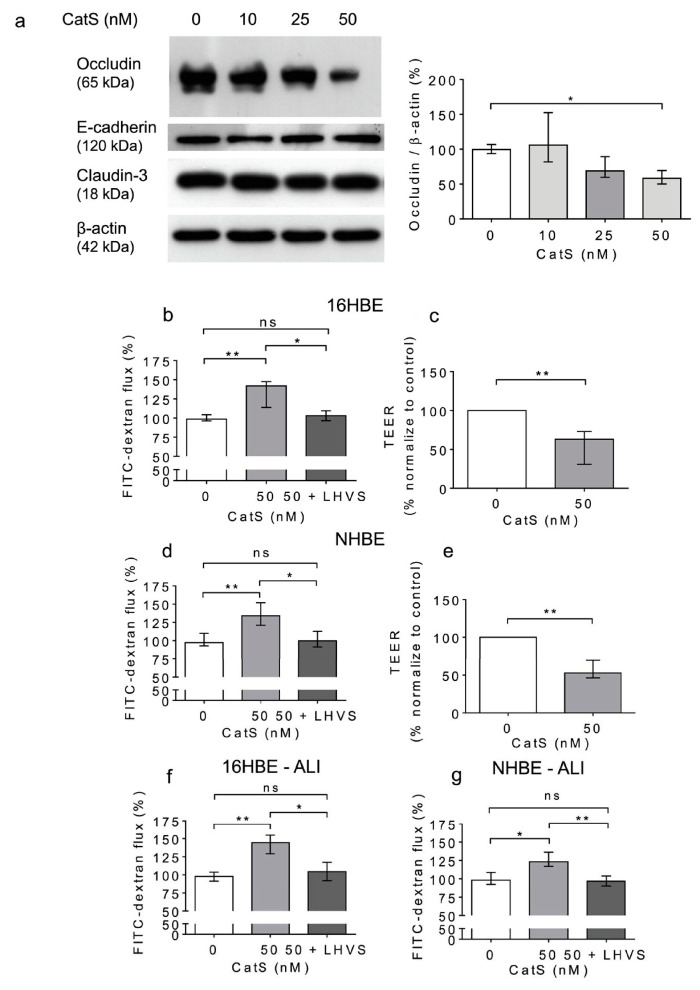
Increased permeability of lung epithelial cells is associated with the CatS-dependent proteolytic damage of occludin. (**a**): 16HBE cells were treated with CatS for 12 h and then lysed. Lysates were submitted to electrophoresis before protein levels of occludin, E-cadherin, and claudin-3 were analyzed by Western blot (β-actin, load control). A representative sample of three independent Western blot experiments is shown. Densitometric analysis of immunoreactive occludin is also shown. (**b**): 16HBE cells were grown on Transwell inserts (submerged culture) and treated with CatS during 12 h. FITC-dextran was added on the upper chamber and fluorescence was measured on the basal chamber. Experiments were performed in presence or absence of LHVS (1 µM). (**c**): Transepithelial electrical resistance (TEER) of 16HBE cells. All the experiments were performed as three independent experiments, in which each were performed in triplicate. Data were normalized to the control experiment. (**d**): NHBE cells were treated by CatS and FITC-dextran permeability assay performed as described above. (**e**): NHBE cells were treated by CatS and TEER was measured. (**f**): FITC-dextran permeability assays were conducted on 16HBE cells that were grown at the air–liquid interface (ALI) and then treated with CatS as described above. (**g**): Identical FITC-dextran permeability assays were conducted on NHBE cells, which were grown at the air–liquid interface (ALI). Data corresponds to the average of five independent experiments. Bars represent median ± quartile. Statistical significance was assessed using non-parametric Kruskal–Wallis (**a**,**b**,**d**,**f**,**g**) or Mann–Whitney U (**c**,**e**) tests (* *p* < 0.05; ** *p* < 0.01). ns: not significant.

Previous results suggested that CatS could degrade adhesion/junctional proteins [33,41,42,62], including occludin (Figure 2). This raises the question of whether CatS could subsequently participate in the increase in cell permeability and thus affect epithelium integrity. Accordingly, we investigated the direct effect of human CatS activity on the permeability of 16HBE cells, a human bronchial epithelial cell line originally obtained from a 1-year-old male and immortalized with SV40 plasmid. CatS was added to culture medium of 16HBE cells. After 12 h of incubation, the cells were harvested, and the lysates were analyzed by Western blot. We did not find any change in immunoreactivity for claudin-3 and E-cadherin (Figure 5a). Conversely, a dose-dependent reduction in the occludin protein level vs. the CatS-untreated 16HBE cells was observed, demonstrating that the decrease in immunoreactive occludin depended on the activity of CatS in the conditioned medium (Figure 5a). Next, we appraised the impact of CatS on the permeability of the 16HBE cells seeded on Transwell inserts, using FITC-dextran as a fluorescence reporter molecule. A conclusive increase in epithelial permeability was observed in the presence of CatS (*p* < 0.01). On the other hand, there was a complete restoration of basal permeability of the 16HBE cells in the presence of LHVS, a specific CatS inhibitor (Figure 5b). These data were corroborated by a cell-based transepithelial electrical resistance (TEER) assay, which demonstrated that the exposure of 16HBE cells to CatS led to decreased TEER (*p* = 0.008, 12 h post-treatment) (Figure 5c).

**Figure 6:**
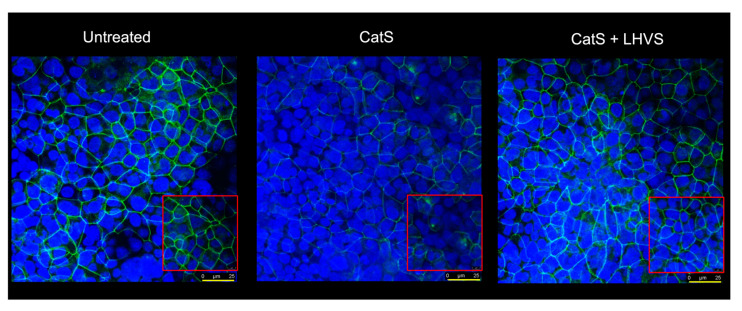
Immunofluorescence analysis. Epithelial 16HBE cells were seeded on Transwell inserts (submerged culture) and treated with CatS as described above for FITC-dextran assays. LHVS (1 µM) was used as control. Cells were exposed to an anti-occludin primary antibody and then incubated with a fluorescein-labeled anti-mouse IgG1 antibody. A representative immunofluorescence image from z-stacks overlaid with Hoechst 33342 nuclear stain of three independent experiments is shown. Scale bar: 25 µm (in yellow). The inserted area (framed: red color) corresponds to an original acquisition zoom (scale bar: 8 µm).

A similar increase in permeability, linked to a drop in transepithelial electrical resistance, was observed following the incubation of normal human bronchial/tracheal epithelial cells (NHBE cells) with CatS (Figure 5d–e). Beside standard (i.e., submerged) cultures, the air–liquid interface (ALI) approach, in which the apical surface of the epithelial cells is exposed to air, is primarily used to mimic in vivo respiratory tract epithelia, since human bronchial/tracheal epithelial cells undergo muco-ciliary differentiation and possess a pseudostratified morphology. Accordingly, ALI cell cultures may provide more robust and physiologically relevant models for validating assays on barrier function and/or epithelial permeability [63]. As reported for the 16HBE and NHBE cells cultured under standard conditions, CatS induced an increase in the permeability of the 16HBE and NHBE cells cultured at an air–liquid interface, which was significantly impaired by the addition of LHVS (Figure 5f–g). Next, considering this variation of epithelial permeability, we analyzed the impact of CatS activity on the tight junctions to further explore the molecular mechanism by which CatS could injure the lung epithelium. Confocal microscopy studies indicated that the immunolabelling and distribution of occludin within tight junctions were drastically altered by CatS (Figure 6).

In the presence of LHVS-inactivated CatS, the fluorescence signal of immunoreactive occludin was recovered, confirming that occludin is a specific target of CatS. Markedly, occludin, which is a 65-kDa tetraspan integral membrane protein, is known to contribute to tight junction stabilization and optimal barrier function (see for review: [56,64]), and its alteration was correlated with permeability changes, making it a likely candidate with respect to regulating epithelial integrity [65]. Taken together, the present data suggest that CatS may weaken lung barrier function by impairing TEER and increasing the permeability of epithelial cells, which is connected to its ability to proteolytically damage tight junction occludin.

### 3.6. Exposure to CSE Induced a Cathepsin S-Dependent Increase in Epithelial Permeability

**Figure 7:**
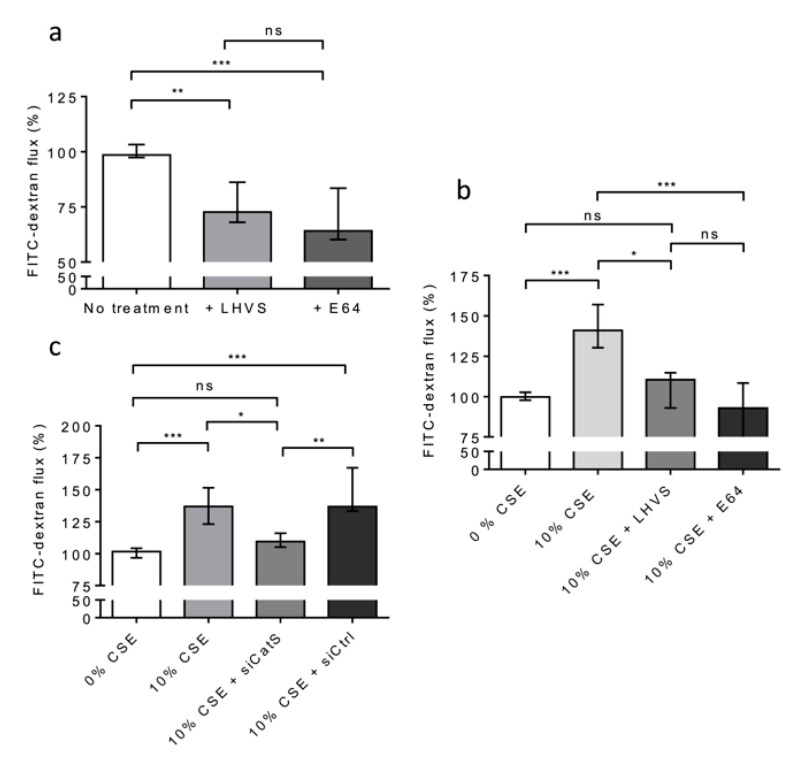
CatS secreted by THP-1 macrophages induced an increase in permeability of lung epithelial cells. (**a**): THP-1 cells (lower culture chamber) were co-cultured with 16HBE cells (seeded on Transwell inserts, upper culture chamber). FITC-dextran permeability assays were performed as described above. LHVS (100 nM) or E64 (1 µM) were used as control. (**b**): Otherwise, THP-1 cells (lower culture chamber), which were co-cultured with 16HBE cells (seeded on Transwell inserts, upper culture chamber), were exposed to CSE. LHVS or E-64 were used as control. FITC-dextran permeability assays were further performed. (**c**): FITC-dextran permeability assays. Transient knockdown of CatS was achieved by transfection of THP-1 cells by specific siRNAs. Normalized data corresponded to the average of three independent experiments. Bars represent median ± quartile. Statistical significance was assessed using Kruskal–Wallis test (* *p* < 0.05; ** *p* < 0.01; *** *p* < 0.001). ns: not significant.

Alternatively, THP-1 cells (situated on 12-well plates of the lower culture chamber) were co-cultured with 16HBE cells (seeded on Transwell inserts placed in the upper culture chamber) and exposed to CSE (10% and for 12 h) at day 6. First, we appraised the basal permeability of the 16HBE cells in the lack of CSE treatment. A highly significant decrease in FITC-Dextran flux was observed in the presence of E-64 (Figure 7a). Similar results were observed in the presence of LHVS, sustaining the notion that the active CatS secreted by untreated, differentiated THP-1 cells [53] increased the basal permeability of the 16HBE cells (Figure 7a). Next, the permeability of the treated 16HBE cells towards FITC-Dextran was dramatically enhanced (*p* < 0.001) following exposure of the THP-1 macrophages to CSE (Figure 7b). On the other hand, the addition of LHVS or E-64 restored the FITC-Dextran flux measured in the absence of CSE. Accordingly, the data suggested that CSE-stimulated expression and the secretion of active CatS by THP-1 cells is associated with the modulation of epithelial permeability. Then, gene silencing of CatS was achieved by the transfection of THP-1 cells by a mixture of four specific siRNAs. The inhibition of transcription was optimal at 48 h, and the transient knockdown (circa 70%) of CatS was validated by Western blot analysis (Figure S4). As a consequence of CatS silencing, the initial impermeability of the 16HBE epithelial cells was recovered and compared to that measured in the lack of CSE treatment (Figure 7c). Conversely, the transfection of macrophages by a scrambled siRNA did not impair the noteworthy increase in FITC-Dextran flux elicited by 10% CSE (*p* < 0.001).

**Figure 8:**
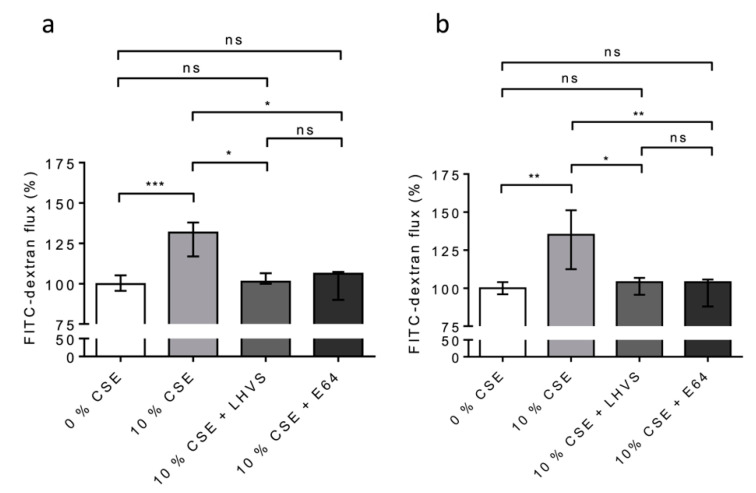
Secreted CatS induced an increase in permeability of lung epithelial cells cultured at the air–liquid interface (ALI). (**a**): Again, THP-1 cells were co-cultured with 16HBE cells; however, this time, the 16HBE cells were cultured at the air–liquid interface. (**b**): NHBE cells (ALI). LHVS or E-64 were used as control. FITC-dextran permeability assays were performed as described above. Normalized data corresponded to the average of five independent experiments. Bars represent median ± quartile. Statistical significance was assessed using Kruskal–Wallis test (* *p* < 0.05; ** *p* < 0.01; *** *p* < 0.001). ns: not significant.

**Figure 9:**
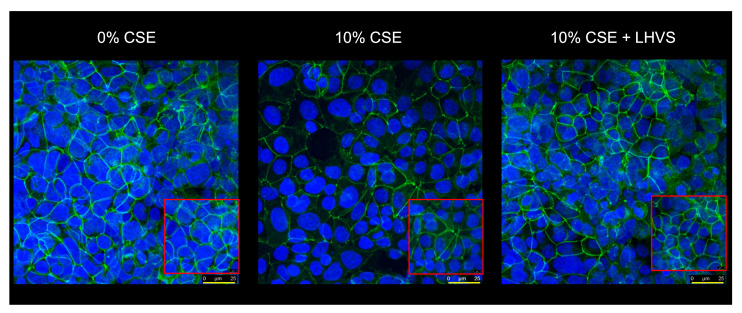
Occludin is a proteolytic target of CSE-induced CatS within tight junctions of lung epithelial cells. As described earlier, 16HBE cells (upper culture chamber) were co-cultured with THP-1 cells (lower culture chamber), which were exposed to CSE (0–10%) for 12 h. LHVS (100 nM) was used as control. After incubation, 16HBE cells were treated with an anti-occludin antibody, as described earlier. Images were acquired in z-stack, with the first and last planes of focus corresponding to a thickness of 1 cell. Representative confocal microscopy images from z-stacks overlaid with Hoechst 33342 (nuclear stain) of three independent experiments are shown. Scale bar: 25 µm (in yellow). The inserted area (framed: red color) corresponds to an original acquisition zoom (scale bar: 8 µm).

We repeated the experiments on the 16HBE (Figure 8a) and NHBE (Figure 8b) cells cultured at an air–liquid interface to confirm/consolidate the previous data. Again, a significant increase in FITC-Dextran flux (16HBE-ALI, *p* < 0.001; NHBE-ALI, *p* < 0.01) was observed following the exposure of the macrophages to CSE, while the addition of both pharmacological inhibitors E-64 and LHVS restored the epithelial barrier function found for the untreated THP-1 cells (Figure 8). Accordingly, the data demonstrate that cigarette smoke triggered the overexpression and secretion of enzymatically active CatS by macrophages, which, in turn, contributed to the impairment of the permeability regulation of lung epithelial cells. Moreover, the drop in the fluorescence signal when using an anti-occludin antibody confirmed that the CSE-driven decrease in epithelial integrity was associated with a loss of immunoreactive occludin (Figure 9). In agreement with confocal microscopy analysis, a similar decrease in immunoreactive occludin was observed by immunoblotting (See Figure S5).

Conversely, the fluorescence signal was regained in the presence of LHVS, which confidently demonstrated that tight junction occludin is a proteolytic target of secreted CatS after the challenge of THP-1 macrophages by nicotine-containing CSE. Consistently, the direct exposure of epithelial cells to CSE had no impact on the amount of immunoreactive occludin, thereby confirming the upstream effect of CSE on the permeability regulation facilitated by tight junctions (data not shown).

## 4. Discussion

Recent findings have evidenced the emerging roles of lysosomal cysteine cathepsins in lung pathophysiology (for review: [17,28,29]). Under particular physiological and pathophysiological settings, cysteine cathepsins may be secreted into the extracellular space [10]. Contrary to other related cysteine cathepsins, extracellular CatS remains stable and active in neutral or slightly alkaline environments [33,40], which corresponds to the mucosal pH of the inflamed airways of smokers [66]. CatS, which is essentially expressed by professional antigen-presenting cells (e.g., dendritic cells, monocytes, and macrophages), displays compelling elastinolytic activity similar to CatV [67]. The dysregulated expression and activity of CatS have been linked to the pathogenesis of lung diseases and pulmonary disease comorbidities via the promotion of inflammation [39,68,69]. In addition, the concentration of extracellular CatS was correlated with the decrease in the respiratory capacity of patients [70]. Elevated CatS levels were found in the broncho-alveolar lavage and plasma fluids of COPD patients, as well as in the lung tissues of smokers with or without COPD [36]. Given its marked elastin-degrading activity, the overexpression of secreted CatS may be harmful, playing a deleterious role in the pathophysiology of COPD and emphysema, as supported by experimental results showing that CatS-deficient mice were more resistant to smoke-induced loss of lung function. However, an essential question arose from this previous research. Despite oxidative stress due to cigarette smoke exposure, how does CatS partially retain its proteolytic activity? We established that the unexpected resistance to major CS oxidants of the nucleophilic Cys of the active site depends on the formation of a reversible sulfenic acid, which can be partially switched by antioxidants [26,37]. On the other hand, cigarette smoke increases the lungs’ epithelial permeability [71], favoring exacerbation phases in emphysema. In addition, considering that the airways of smokers have a weakly basic/neutral (~7.2–7.3) mucosal pH unlike normal healthy airway mucosa (slightly acidic pH averaging 6.6) [66], one can wonder if CatS, via the proteolytic targeting of junctional proteins, could participate in the modulation of the permeability of lung epithelial cells.

In an earlier study, we demonstrated that CatS activity was significantly higher in the lung tissue of smokers and correlated positively with smoking history [36]. Here, using lung biopsies issued from the same cohort, we achieved a systematic immunochemical analysis of representative tight junction proteins. The experimental data show that the expression level of occludin is reduced in the lung tissue from smokers. Our Spearman analysis revealed that the occludin level correlated negatively with the severity of the disease (GOLD stages) and with the smoking status (expressed as pack-years). Additionally, the negative correlation between immunoreactive occludin and CatS activity demonstrated that 65-kDa occludin could be a specific target of the protease. Of interest, murine claudin-1 from the tight junctions of intestinal epithelium was proposed as a target of closely related cathepsin L [57]. Herein, no statistically relevant relationships were found between the expression levels of both human claudins-1 and -3 and smoking status, GOLD stages, pack-years, and CatS activity, suggesting that tight junction claudins are not CatS targets in human lungs.

**Figure 10:**
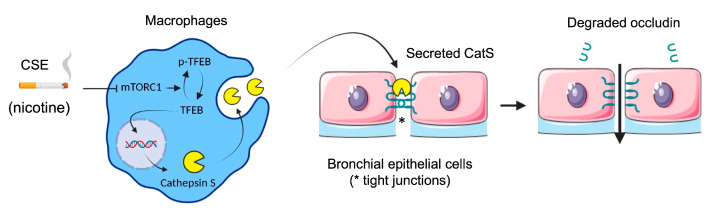
Proposed mechanism of cigarette smoke-induced increase in lung epithelial cell permeability (synthetic graphic). Cigarette smoke triggers upregulation of CatS by macrophages through the mTOR/TFEB signaling pathway. CatS overexpression was driven by nicotine, a major chemical of cigarette smoke. Consequently, secreted active CatS could proteolytically damage occludin, leading to impaired integrity of the epithelial barrier. Figure prepared with BioRender (https://biorender.com/, accessed on 2 April 2021).

Next, we evaluated the impact of oxidative stress induced by the exposure of macrophages to CSE according to differentiated THP-1 macrophages that secrete catalytically active CatS [53]. Consistently, the CatS promoter contains a functional IFN-γ-stimulating response element that triggers CatS transcription by binding to IRF-1; in addition, inflammatory interleukins (IFN-γ or IL-18) may stimulate CatS expression, while IL-10 represses its production [33]. Complementary signaling pathways (e.g., STAT or PI3K/Akt) may be involved in the transcriptional regulation of CatS (for review: [48]). Here, in contrast to what was observed with CSE-exposed primary human bronchial cells, we did not uncover activation of P2X7 receptors, which, in turn, may drive CatS upregulation [36]. Yet, CSE induced the inhibition of mTOR and the subsequent dephosphorylation of TFEB, which is involved in the response to stress conditions and is primarily known as a regulator of autophagy-lysosomal biogenesis [60]. The involvement of mTOR was indicated by its pharmacological inhibition by rapamycin. Moreover, the exposure of THP-1 cells to CSE led to a significant increase in mRNA levels, protein levels, and the secretion of active CatS, depending on the mTOR signaling pathway, which is in agreement with previous analyses on the transcriptionally TFEB-dependent regulation of CatS [19,69]. In line with our investigations, another group demonstrated that nicotine, a foremost chemical of cigarette smoke, upregulated CatS expression by VSMCs and that TFEB directly binds to the CatS promoter [52]. As recently reported by Turk and colleagues, the over-expression of cysteine cathepsins (including CatS) arises as a regular consequence of TFEB activation [72]. Here, the incubation of THP-1 macrophages by nicotine elicited an increase in both CatS mRNA and protein expression levels as well as CatS activity, and a decrease in phosphorylated TFEB, which was comparable to that observed in the presence of 10% CSE or rapamycin. Accordingly, we propose that, following the exposure of macrophages to CSE, the nicotine-driven upregulation of CatS occurs through the mTOR/TFEB signaling pathway, as observed for VSMCs (Figure 10). More probably, CSE via interactions between nicotine and mTOR, together with the nuclear shuttling of TFEB from the cytoplasm to the nucleus, may stimulate the fusion of lysosomal and plasma membranes for subsequent CatS exocytosis and the release of active CatS [73].

Then, we assessed the consequences of the addition of ectopic CatS on the epithelial barrier integrity of lung 16HBE and NHBE cells. We observed a significant increase in FITC-Dextran flux, while transepithelial electrical resistance decreased, indicating that CatS has a deleterious impact on cellular permeability. However, a possible bias of standard cell cultures (i.e., submerged lung epithelial cells) is that cells fail to undergo muco-ciliary differentiation and gain baso-apical polarity. The air–liquid interface (ALI) approach allows for the mimicry of respiratory tract epithelia since the basal surface of epithelial cells is in contact with the liquid culture medium, whereas the apical surface is exposed to air [63]. So, ALI cells undergo muco-ciliary differentiation and pseudostratified morphology of physiological relevance, providing a robust model with which to confirm and validate previous analysis of epithelial permeability. As initially observed under standard conditions, an analogous increase in the permeability of 16HBE and NHBE cells cultured under ALI conditions was measured after the addition of CatS. Moreover, LHVS significantly impaired the change in permeability, certifying that the degree of damage to the epithelial integrity depends on the catalytic activity of the enzyme. Likewise, both Western blot and fluorescence confocal microscopy investigations suggested that occludin is a CatS target, for which proteolytic degradation contributed to the impairment of the tight junction stabilization and optimal barrier function [56,64].

Afterward, the 16HBE cells were co-cultured with THP-1 cells, which had been exposed to CSE at day 6. The FITC-Dextran flux of the epithelial cells was dramatically enhanced following the exposure of the THP-1 macrophages to CSE. Conversely, both the addition of LHVS and CatS silencing of THP-1 cells by specific siRNAs identically restored the FITC-Dextran flux measured in the absence of CSE, demonstrating that active CatS secreted by THP-1 macrophages specifically increased the permeability of 16HBE cells. The results were further validated by alike experiments but conducted with 16HBE and NHBE cells cultured at an air–liquid interface. The findings confirmed that cigarette smoke triggers the overexpression and secretion of enzymatically active CatS by macrophages, which, in turn, contribute to the damage of barrier function of lung epithelial cells. Again, as observed by fluorescence confocal microscopy, the loss of CSE-driven impermeability was associated with a strong decrease in the occludin level within tight junctions, which was regained by the pharmacological inhibition of CatS. Alongside the current identification of occludin as a CatS target, it was noticed that a decorin-derived peptide specifically released by CatS was identified in the serum of patients with COPD and proposed as a potential COPD biomarker [74]. Furthermore, a study supported that Der p1, a cysteine protease from house dust mite (*Dermatophagoides pteronyssinus*) allergens, cleaved occludin and led to an increase in epithelial permeability [75]. Hence, such a proteolytic mechanism could favor allergen delivery across lung and nasal epithelial barriers in asthmatic and allergic rhinitis patients [76]. Accurately, CatS has been associated with asthma pathophysiology [77,78], and CatS inhibitors have been evaluated (preclinical trials) for the treatment of bronchial asthma [79]. Herein, we propose that cysteine cathepsin S plays an essential role in the homeostasis of lung epithelia and can have deleterious effects under inflammatory conditions. Nevertheless, the contribution of supplementary proteolytic enzymes, including other cysteine proteases and metalloproteinases, to the CS-dependent injury of epithelium cannot be disregarded at this stage [64,80].

## 5. Conclusions

To summarize, an immunoreactive amount of occludin, a key protein involved in the stability and cohesion of epithelial tight junctions, was significantly decreased in t lung tissue from smokers vs. non-smokers. The occludin level was negatively correlated with smoking history (number of pack-years), COPD grades, and CatS activity. Accordingly, we hypothesized that CatS activity could be involved in lung epithelial barrier alteration via the proteolytic damage of occludin. Otherwise, we suggested that CSE triggered the upregulation of CatS by macrophages through the mTOR/TFEB signaling pathway. Subsequently, the secreted CatS could target occludin within tight junctions of lung epithelial cells. The proteolytic CatS-dependent damage of occluding most likely induced the destabilization of tight junctions associated with a reduction in cohesive properties, as supported by an impaired TEER and a notable weakening of epithelium impermeability. Taken together with its prominent role in elastic fiber destruction during emphysema, the present data emphasize the emerging role of CatS in smoking-related lung diseases and consequently strengthen the therapeutic relevance of targeting CatS in emphysema and COPD patients.

## Figures and Tables

**Table 1:** Clinical characteristics of patients.

	Non-COPD		COPD		
			GOLD I	GOLD II	GOLD III
Smoking status	NS	S	S	S	S
No. of subjects	6	6	6	5	5
Age, years (SD)	66 (12)	58 (8)	70 (12)	62 (7)	70 (10)
Pack-years (SD)	0	32 (20)	37 (26)	56 (25)	51 (20)
FEV1, % predicted (SD)	90 (21)	97 (10)	93 (21)	67 * (5)	41 * (6)

Values are presented as n or as means (SD). Smoking status is noted as follows: NS, never-smoker; S, smoker. Mean age of each group was compared using Kruskal–Wallis test; no significant differences were found. FEV1, forced expiratory volume in 1 s (% predicted). The GOLD (Global Initiative for Chronic Obstructive Lung Disease) system categorizes airflow limitation into stages: GOLD I, mild stage with a FEV1 ≥ 80%; GOLD II, moderate stage (50% ≤ FEV1 < 80% predicted); Gold III, severe stage (30% ≤ FEV1 < 50% predicted). FEV1 data were compared using Kruskal–Wallis test (* *p* < 0.05).

## Data Availability

Data generated during this study are included in the article and Supplementary Materials.

## Supplementary Materials

The following supporting information can be downloaded at: https://www.mdpi.com/article/10.3390/antiox12010005/s1, Supplementary Material and Methods.pdf; Figure S1. Claudin-1 expression in lung specimen; Figure S2. Claudin-3 expression in lung specimen; Figure S3. Analysis of the cytotoxic effects of cigarette smoke extract (CSE), nicotine, and rapamycin on cell viability; Figure S4. Western blot analysis of the transient knockdown of cathepsin S by RNA interference; Figure S5. Decrease in tight junction occluding following CSE-driven secretion of cathepsin S. [81].
