# Supplementary material for: Cleavage of Occludin by Cigarette Smoke-Elicited Cathepsin S Increases Permeability of Lung Epithelial Cells
Source: Antioxidants (Basel). 2022 Dec 21;12(1):5. doi: 10.3390/antiox12010005 (PMC9854811; doi:10.3390/antiox12010005)
Supplement: Supplementary file 1 [file antioxidants-12-00005-s001.zip › antioxidants-2042357-supplementary.pdf]

Details of the materials and employed methods are provided in the Supplemental Material online.

Cleavage of occludin by cigarette smoke-elicited cathepsin S increases permeability of lung epithelial cells

Paul Bigot[1,2], Simon Chesseron[1,2], Ahlame Saidi[1,2], Damien Sizaret[1,3], Christelle Parent[1,4], Agnès Petit-Courty[1,2], Yves Courty[1,2], Fabien Lecaille[1,2] and Gilles Lalmanach[1,2 *]

1 University of Tours, Tours, France
2 INSERM, UMR1100, Research Center for Respiratory Diseases (CEPR), Team "Proteolytic Mechanisms in Inflammation", Tours, France
3 The University Hospital Center of Tours, Pathological Anatomy and Cytology, Tours, France
4 INSERM, UMR1100, Research Center for Respiratory Diseases (CEPR), Team "Aerosol therapy and Biotherapeutics for Respiratory Diseases", Tours, France
* Correspondence: e-mail: gilles.lalmanach@univ-tours.fr; Tel.: (+33) 2 47 36 61 51

Supplemental material: Table of contents

- Page 2 - Page 5: Supplementary Materials and Methods
- Page 6: Figure S1
- Page 7: Figure S2
- Page 8: Figure S3
- Page 9: Figure S4
- Page 10: Figure S5

Supplemental Materials and Methods

Inhibitors, chemicals, and recombinant protein

Morpholinourea-leucinyl-homophenylalanine-vinyl-sulfone phenyl (LHVS) was a kind gift from Prof. James H McKerrow (Skaggs School of Pharmacy and Pharmaceuticals Sciences, University of California, San Diego, CA, USA). Pefabloc, EDTA, S-methyl thiomethanesulfonate (MMTS), pepstatin A, and E-64 were purchased from Sigma-Aldrich (Saint Quentin Fallavier, France). Rapamycin and rat-type I collagen were purchased from Thermo Fisher scientific (Illkirch-Graffenstaden, France). Recombinant E-cadherin, claudin-3, and occludin were supplied by Bio-Techne SAS (Noyal-Chatillon-sur-Seiche, France), while recombinant Jam-B was purchased from Abcam (Paris, France). AZ 11645373 (a.k.a 3-[1-[[(3'-Nitro[1,1'-biphenyl]-4-yl)oxy]methyl]-3-(4-pyridinyl)propyl]-2,4-thiazolidinedione), a P2X7 antagonist, was procured from Bio-Techne SAS. Nicotine, Phorbol 12-myristate 13-acetate (PMA), and 4 kDa FITC-Dextran were supplied by Sigma-Aldrich.

Human cathepsin S

Recombinant cathepsin S (CatS) was produced according to [1]. The activity buffer of CatS was 0.1 M sodium acetate buffer, with pH 5.5, containing 5 mM DTT, 1 mM EDTA, and 0.01% Brij 35. CatS activity was recorded using Z-Leu-Arg-AMC as a substrate (excitation wavelength = 350 nm, emission wavelength = 460 nm) (Bachem, Voisin-le-Bretonneaux, France). Fluorescence release was monitored using a Spectramax Gemini spectrofluorometer (Molecular Devices, Saint Grégoire, France). The active concentration of CatS was determined by titration with E-64, a broad-spectrum inhibitor of cysteine cathepsins [2].

Measurement of cathepsin S activity in lung tissue samples

CatS activity was determined according to a previous report [3]. Briefly, aliquots (corresponding to 5 µg of total protein) were preincubated in 100 mM sodium-phosphate buffer pH 7.4 (1 h, 37°C) to inactivate other cysteine cathepsins under neutral conditions. Then, CatS activity was measured at 37°C using Z-Leu-Arg-AMC (20 µM, Bachem) as substrate in 0.1 M sodium acetate buffer with pH of 5.5 and containing 5 mM of DTT, 1 mM of EDTA, and 0.01% Brij 35 in the presence of an increasing concentration of E-64 (0-500 nM). Triplicate assays were repeated as three independent experiments. Controls were created using the CatS irreversible inhibitor LHVS (0-500 nM).

Western blotting

Rabbit polyclonal antibodies directed against human claudin-1 and human claudin-3 and mouse monoclonal antibodies directed against human occludin and human E-cadherin came from Invitrogen (Carlsbad, CA, USA). Rabbit polyclonal antibodies directed against human Jam-B were purchased from Abcam. Goat anti-human CatS antibodies were purchased from R&D Systems (Bio-Techne SAS). Rabbit polyclonal anti-TFEB and monoclonal anti-phospho-TFEB (Ser211) antibodies came from Cell Signaling Technology (Saint-Cyr-L'Ecole, France). Mouse monoclonal antibodies directed against human β-actin were provided by Sigma-Aldrich. Secondary polyclonal anti-goat, anti-rabbit, and anti-mouse antibodies conjugated with horseradish peroxidase were provided by Sigma-Aldrich. Protein samples were prepared in Laemmli buffer, boiled for 5 min, and then loaded onto SDS-PAGE (under reducing conditions). The separated proteins were transferred to a nitrocellulose Hybond-ECL membrane (Amersham Biosciences, Buckinghamshire, UK), which was saturated with PBS, 0.1% Tween 20, and 5% non-fat powdered milk (PBS-T), and then incubated with the primary antibody (1 :1000 in PBS-T) overnight at 4°C under agitation. Following washing procedure, the secondary peroxidase-conjugate antibody (1:5000 in PBS-T) was added and left for 1 h at room temperature. Finally, proteins were visualized by chemiluminescence (ECL Plus Western Blotting Detection system, Amersham Biosciences). Bands were quantified by densitometric analysis using the ImageJ software (NIH, Bethesda, MD, USA) and data were normalized to β-actin signal [4] or TFEB signal.

Silencing of cathepsin S by RNA interference and quantitative real-time polymerase chain reaction (RT-qPCR)

Specific, predesigned small interfering RNAs for CatS (siCatS) and scrambled siRNA were obtained from Qiagen SA (Courtaboeuf, France). The target sequences were Hs_CTSS_1, AAGGATATATTCGGATGGCAA; Hs_CTSS_2, AGGAATCTAATTATATCGAAA; Hs_CTSS_3, CTGCCACATGTTCAAAGTACA; and Hs_CTSS_5, CTCATACGATCTGGGCATGAA; THP-1 macrophages were transfected with a cocktail of the four SiRNA (12.5 nM) in basal medium using HiPerFect transfection reagent (Qiagen SA) for 6 h. Total RNA was extracted and purified using the RNeasy Mini kit (Qiagen SA). Total RNA (0.3 µg) was reverse-transcribed using the RevertAid Moloney murine leukemia virus reverse transcriptase (RevertAid M-MuLV; Fermentas, Waltham, MA, USA).

Reduction of transcripts was determined by quantitative real time PCR (LightCycler 480 system, Roche Diagnostics GmbH, Mannheim, Germany) using PowerUP SYBR Green fluorescent mix (Thermo Fisher Scientific). Sequences of PCR primers were as follows: 5'GCTTCACAACCTGGAGCATTC3' (sense) and 5'GGCAATATCCGATTAGGGTTTGA3' (antisense) for CatS [5], and 5' CATGTACGTTGCTATCCAGGC3' (sense) and 5'CTCCTTAATGTACGCACGAT3' (antisense) for human β-actin. For quantification of relative expression levels, the ∆∆Ct method was used (normalization gene: Human β-actin).

Exposure of THP1 cells to cigarette smoke extract (CSE)

Human THP-1 monocytic cell line (ATCC, VA, USA) was provided by LGC Promochem (Molsheim, France). Cells were cultured in 1 volume of DMEM supplemented with glutamate and 1 volume of RPMI-1640 Glutamax I, containing 1.5 g/L sodium bicarbonate, 4.5 g/L glucose, 10 mM HEPES, 1 mM sodium pyruvate, and 0.05 mM 2-β-mercaptoethanol (37°C, 5% CO2). The medium was enriched with 10% endotoxin-free heat-inactivated fetal calf serum (FCS) and 50 U/mL of penicillin/streptomycin (Fisher Scientific, Waltham, MA, USA). Briefly, THP-1 monocytes (1.10^6 cells) were seeded onto 6-well plates and differentiated by addition of 162 nM PMA at day 0, as previously reported [6]. At day 6, which corresponds to the maximal secretion of cathepsins [7], macrophages were exposed to CSE (0-10%) for 12 h. Then, culture medium was withdrawn in a preservative buffer (0.1 M sodium acetate buffer, pH 5.5, containing 0.5 mM Pefabloc, 0.5 mM EDTA, 1 mM S-methyl thiomethanesulfonate, and 0.04 mM pepstatin A) and stored at -80°C until further studies [8,9]. A cell viability assay (CellTiter 96® AQueous One Solution Cell Proliferation Assay, MTS kit, Promega, Madison, WI, USA) was conducted to examine potential cytotoxic effects of CSE. Absorbance was measured at 490 nm (VersaMAx Microplate Reader, Molecular Devices). MTS assay results were normalized using the proliferation rate of THP-1 cells cultured in the absence of CSE as a reference (control value defined as 100%). Alternatively, culture media of THP-1 cells were concentrated 50-fold (Vivaspin concentrator tube, exclusion limit 2,000, Sartorius AG, Göttingen, Germany) before adding an equivalent volume of 0.1M HEPES buffer, pH 7.4, EDTA 1mM, Brij35 0.01%, and 10 mM DTT. After one hour of incubation to inactivate cysteine cathepsins except CatS, its activity was measured in the assay buffer at 37°C, using Z-Leu-Arg-AMC (20 µM) as substrate as described elsewhere [10]. Data were normalized (control value in absence of CSE treatment defined as 100%). Same experiments were repeated in the presence of rapamycin (50 nM) [11], AZ 11645373 (P2X7 antagonist, 1 µM) [12], or nicotine (5 µM). All the experiments were performed as three independent experiments, with each performed in triplicate.

Hydrolysis of junction proteins (in vitro assays)

After preincubation (5 min, 37°C) in 0.1 M sodium acetate buffer, at pH 5.5, and containing 1 mM EDTA, 0.01% Brij 35, and 2 mM DTT, a constant amount of recombinant human occludin (100 ng) was mixed with increasing amounts of CatS (enzyme-occludin molar ratios: 1:625 to 1:1) and incubated for 1 h at 37°C in cell culture medium (final volume = 15 µL). A control experiment was carried out in the presence of E-64 (1 µM). Reaction was stopped by adding SDS sample buffer; then, the sample was boiled and subjected to Western blot analysis, in which a monoclonal anti-occludin antibody was employed (Invitrogen). Same experiments were repeated in the presence of E-cadherin (30 ng), claudin-3 (10 ng), and Jam-B (10 ng).

Exposure of human bronchial epithelial cells to CatS

16HBE human bronchial epithelial cells (generously provided by Prof. Dieter C. Gruenert, University of California, San Francisco, CA, USA) were cultured in 1 volume of DMEM supplemented with glutamate and 1 volume of RPMI-1640 Glutamax I, containing 1.5 g/L sodium bicarbonate, 4.5 g/L glucose, 10 mM HEPES, 1 mM sodium pyruvate, and 0.05 mM 2-β-mercaptoethanol (37°C, 5% CO2). The medium was enriched with 10% endotoxin-free heat-inactivated fetal calf serum (FCS) and 50 U/mL penicillin/streptomycin. The $7.5.10^4$/cm² 16HBE cells were seeded onto 6-well plates and grown at 95% confluence. FCS was removed from the media (one day before experiment). Exogenous CatS (0-50 nM) was pre-incubated in 0.1 M sodium acetate buffer, with pH 5.5, and containing 1 mM EDTA, 0.01% Brij 35, and 15 µM DTT (5 min, 37°C); then, it was added to FCS-free culture medium of 16HBE cells. After 12 h incubation, cells were harvested in RIPA buffer. Cell lysates and protein extraction were performed as described above. Occludin, E-cadherin, and claudin-3 were further analyzed by Western blot analysis.

Permeability and transepithelial electrical resistance assays of bronchial epithelial cells

$7.5.10^4$/cm² 16HBE cells were seeded on Transwell inserts (3.0 µm pore, Thermo Scientific). FCS was removed from the medium (one day before experiment). When cells reached confluence, CatS (0-50 nM), previously activated in 0.1 M phosphate buffer, at pH 7.4, and with 15 µM DTT (5 min, 37°C), was added to the conditioned FCS-free medium. After 12 h of incubation, the culture medium was renewed, and FITC-Dextran (2 mg/mL) was added in the upper Transwell chamber. After 20 min, culture medium of the lower chamber was

collected (100 µL) and fluorescence was measured (λex = 485nm, λem = 520nm). All the experiments were performed in triplicate and made as three or five independent experiments. Data were normalized to the control condition without treatment. In addition, transepithelial electrical resistance (TEER, Ω.cm²) was determined according to manufacturer's instructions (volt-ohmmeter EVOM2, World Precision Instrument, Hertfordshire, UK). Briefly, measurements were performed after adding PBS to the basal and apical side of epithelial cells. Resistance control measurements were assessed with a cell-free Transwell chamber. All the experiments were performed as three independent experiments, with each performed in triplicate. Data were normalized to the control experiment. Alternatively, experiments were repeated using normal human bronchial/tracheal epithelial cells (NHBE cells), which were supplied by Lonza (Basel, Switzerland). Briefly, 5.10^4 NHBE cells were seeded on Transwell inserts (24-well plate previously coated with rat type I collagen) were cultured in B-ALI growth medium (Lonza). Measurements of permeability (FITC-Dextran) and TEER were performed once confluence was reached.

Air–liquid interface (ALI) cell culture of bronchial epithelial cells

According to the manufacturer's instructions, 5.10^4 NHBE cells (Lonza) were seeded on Transwell inserts (24-well plate) previously coated with type I collagen. Cells were first cultured (submerged conditions) in B-ALI growth medium (Lonza). After 3 days, B-ALI growth medium was removed from the apical side. Then, epithelial cells were cultured in B-ALI differentiation medium (Lonza), which was renewed every two days until day 21. ALI cultures of 16HBE cells were performed similarly, except that cells were seeded at $7.5.10^4$ /cm² and cultured in 1 volume of DMEM supplemented with glutamate and 1 volume of RPMI-1640 Glutamax I, containing 1.5 g/L sodium bicarbonate, 4.5 g/L glucose, 10 mM HEPES, 1 mM sodium pyruvate, and 0.05 mM 2-β-mercaptoethanol, which was enriched with 10% FCS and 50 U/mL penicillin/streptomycin. Transepithelial electrical resistance was monitored once a week. FCS was removed from the medium (one day before experiment). Following addition of CatS (0-50 nM) to the medium, permeability assays (FITC-Dextran, 2 mg/mL) were performed at day 21. All the experiments were performed in triplicate and made as three or five independent experiments. Data were normalized to the control condition without treatment.

Coculture of THP-1 macrophages and epithelial cells

The 1.10^5/cm² THP-1 monocytes were seeded onto 12-well plates and differentiated into macrophages by adding 162 nM PMA at day 0 [6]. In parallel, $7.5.10^4$ /cm² 16HBE cells were seeded on Transwell inserts (day 3). At day 5, inserts containing the 16HBE cells were placed in the upper culture chamber above 12-well plates seeded by THP-1 macrophages, while FCS was removed from the medium. At day 6, which corresponds both to the differentiation peak of THP-1 macrophages and to the confluence of epithelial cells, 10% CSE or 10% ethanol (control) was added to the FCS-free culture medium for 12 h. The culture medium was renewed before adding FITC-Dextran (2 mg/mL) in the upper Transwell chamber. After 20 min incubation, the culture medium of the lower chamber was collected, and epithelial permeability of 16HBE cells to FITC-Dextran was measured. Same experiments were repeated in the presence of pharmacological inhibitors, LHVS (100 nM), or E-64 (1 µM). Moreover, supplementary analysis was performed by a transient invalidation of CatS by small interfering RNAs. THP-1 macrophages were transfected at day 4 for 6 h with a cocktail of four specific siRNAs for CatS (12.5 nM) or a scrambled predesigned small interfering RNA (Qiagen SA), as described above, prior to the coculturing of 16HBE epithelial cells and THP-1 macrophages. Experiments were repeated with 16HBE cells cultured at the air–liquid interface. Inserts containing 16HBE-ALI cells were placed in the upper chamber above THP-1 macrophages, five days after PMA-induced differentiation. Similar experiments were performed with NHBE-ALI cells cultured at the air–liquid interface in 1 volume of B-ALI differentiation medium and 1 volume of RPMI-1640 Glutamax I, containing 1.5 g/L sodium bicarbonate, 4.5 g/L glucose, 10 mM HEPES, 1 mM sodium pyruvate, and 0.05 mM 2-β-mercaptoethanol. All the experiments were performed in triplicate and made as three or five independent experiments. Data were normalized to the control condition without treatment.

Immunofluorescence analysis

The 16HBE cells were seeded on Transwell inserts and treated with CatS (0-50 nM), as described above for permeability assays. After incubation, cells were washed and fixed with paraformaldehyde (3.7%). Non-specific sites were blocked with PBS-BSA 3% for 1 h at room temperature. Afterwards, Cells were exposed to an anti-occludin primary antibody (Invitrogen, 1:50 in PBS-BSA 3%) overnight at 4°C, and then incubated with a fluorescein-labeled anti-mouse IgG1 antibody (Thermo Fisher scientific; 1:1000) for 1 h at room temperature. Cells were counterstained with Hoechst 33342 (Thermo Fisher scientific) for 10 min at room temperature. Transwell membranes were released and mounted using ProLong Diamond antifade (Thermo Fisher scientific). Immunolabelling of occludin was assessed by Olympus FV 500 confocal laser-scanning microscope (Olympus France, Rungis, France). Images were acquired in z-stack, with the first and last planes of focus corresponding to a thickness of 1 cell.

References:

1. Sage, J.; Mallèvre, F.; Barbarin-Costes, F.; Samsonov, S.A.; Gehrcke, J.-P.; Pisabarro, M.T.; Perrier, E.; Schnebert, S.; Roget, A.; Livache, T.; et al. Binding of Chondroitin 4-Sulfate to Cathepsin S Regulates Its Enzymatic Activity. Biochemistry 2013, 52, 6487–6498, doi:10.1021/bi400925g.
2. Barrett, A.J.; Kembhavi, A.A.; Brown, M.A.; Kirschke, H.; Knight, C.G.; Tamai, M.; Hanada, K. L-Trans-Epoxysuccinyl-Leucylamido(4-Guanidino)Butane (E-64) and Its Analogues as Inhibitors of Cysteine Proteinases Including Cathepsins B, H and L. Biochem. J. 1982, 201, 189–198, doi:10.1042/bj2010189.
3. Naudin, C.; Joulin-Giet, A.; Couetdic, G.; Plésiat, P.; Szymanska, A.; Gorna, E.; Gauthier, F.; Kasprzykowski, F.; Lecaille, F.; Lalmanach, G. Human Cysteine Cathepsins Are Not Reliable Markers of Infection by Pseudomonas Aeruginosa in Cystic Fibrosis. PLoS One 2011, 6, e25577, doi:10.1371/journal.pone.0025577.
4. Kasabova, M.; Joulin-Giet, A.; Lecaille, F.; Gilmore, B.F.; Marchand-Adam, S.; Saidi, A.; Lalmanach, G. Regulation of TGF-Β1-Driven Differentiation of Human Lung Fibroblasts: Emerging Roles of Cathepsin B and Cystatin C. J. Biol. Chem. 2014, 289, 16239–16251, doi:10.1074/jbc.M113.542407.
5. Veillard, F.; Saidi, A.; Burden, R.E.; Scott, C.J.; Gillet, L.; Lecaille, F.; Lalmanach, G. Cysteine Cathepsins S and L Modulate Anti-Angiogenic Activities of Human Endostatin. J Biol Chem 2011, 286, 37158–37167, doi:10.1074/jbc.M111.284869.
6. Hervé-Grépinet, V.; Veillard, F.; Godat, E.; Heuzé-Vourc’h, N.; Lecaille, F.; Lalmanach, G. Extracellular Catalase Activity Protects Cysteine Cathepsins from Inactivation by Hydrogen Peroxide. FEBS Lett. 2008, 582, 1307–1312, doi:10.1016/j.febslet.2008.03.007.
7. Wartenberg, M.; Saidi, A.; Galibert, M.; Joulin-Giet, A.; Burlaud-Gaillard, J.; Lecaille, F.; Scott, C.J.; Aucagne, V.; Delmas, A.F.; Lalmanach, G. Imaging of Extracellular Cathepsin S Activity by a Selective near Infrared Fluorescence Substrate-Based Probe. Biochimie 2019, 166, 84–93, doi:10.1016/j.biochi.2019.03.013.
8. Perdereau, C.; Godat, E.; Maurel, M.-C.; Hazouard, E.; Diot, E.; Lalmanach, G. Cysteine Cathepsins in Human Silicotic Bronchoalveolar Lavage Fluids. Biochim. Biophys. Acta 2006, 1762, 351–356, doi:10.1016/j.bbadis.2005.10.005.
9. Serveau-Avesque, C.; Martino, M.F.-D.; Hervé-Grépinet, V.; Hazouard, E.; Gauthier, F.; Diot, E.; Lalmanach, G. Active Cathepsins B, H, K, L and S in Human Inflammatory Bronchoalveolar Lavage Fluids. Biol Cell 2006, 98, 15–22, doi:10.1042/BC20040512.
10. Galibert, M.; Wartenberg, M.; Lecaille, F.; Saidi, A.; Mavel, S.; Joulin-Giet, A.; Korkmaz, B.; Brömme, D.; Aucagne, V.; Delmas, A.F.; et al. Substrate-Derived Triazolo- and Azapeptides as Inhibitors of Cathepsins K and S. Eur J Med Chem 2018, 144, 201–210, doi:10.1016/j.ejmech.2017.12.012.
11. Ni, H.; Xu, S.; Chen, H.; Dai, Q. Nicotine Modulates CTSS (Cathepsin S) Synthesis and Secretion Through Regulating the Autophagy-Lysosomal Machinery in Atherosclerosis. Arterioscler Thromb Vasc Biol 2020, 40, 2054–2069, doi:10.1161/ATVBAHA.120.314053.
12. Andrault, P.-M.; Schamberger, A.C.; Chazeirat, T.; Sizaret, D.; Renault, J.; Staab-Weijnitz, C.A.; Hennen, E.; Petit-Courty, A.; Wartenberg, M.; Saidi, A.; et al. Cigarette Smoke Induces Overexpression of Active Human Cathepsin S in Lungs from Current Smokers with or without COPD. Am J Physiol Lung Cell Mol Physiol 2019, 317, L625–L638, doi:10.1152/ajplung.00061.2019.

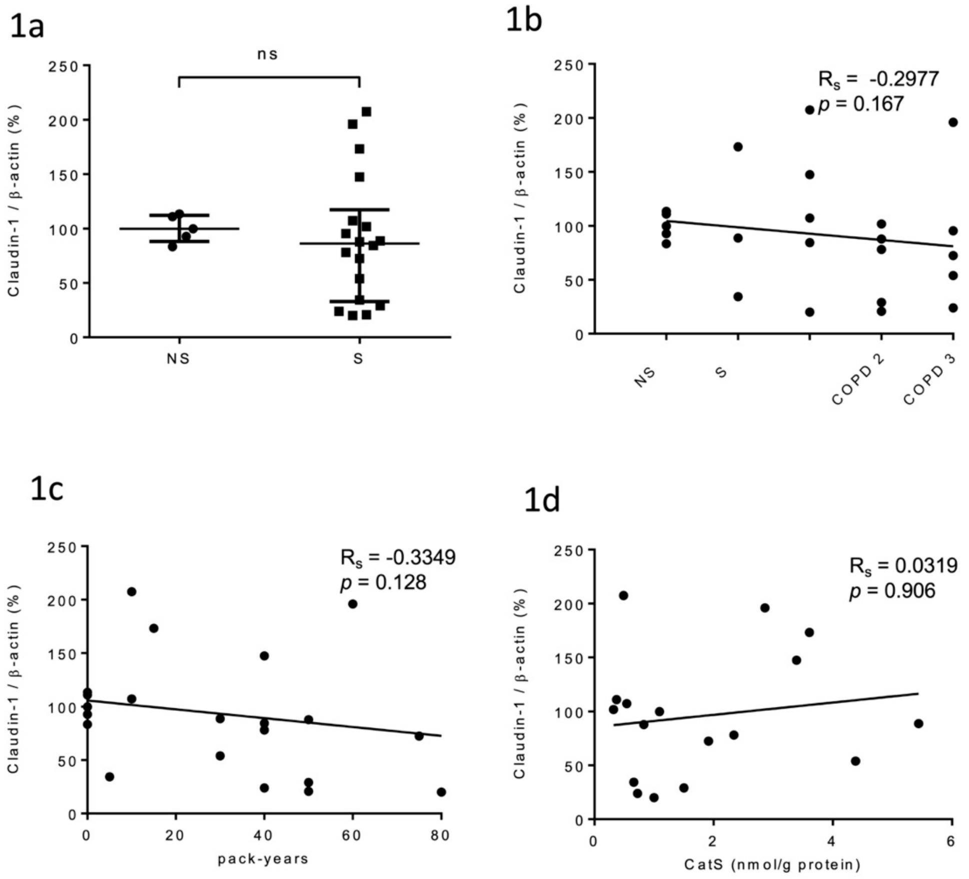

Supplementary Figure S1: Claudin-1 expression in lung specimen

a: The expression level of immunoreactive claudin-1 was analyzed by Western blot. Densitometric analysis of claudin-1 was performed (ImageJ software) using β-actin (load control) for normalization. Bars represent median ± quartile. No statistical significance (ns) was found (Mann–Whitney test).

b: Correlation between the expression level of claudin-1 and patient status.

c: Correlation between the expression level of claudin-1 and the smoking history (packs/year). Correlations were determined by linear regression and expressed by Spearman coefficient (R_s) and levels of significance (p).

d: Correlation between CatS activity and the expression level of claudin-1. CatS concentration (tissue lysate, 10µg of total protein) was determined with E-64, using Z-Leu-Arg-AMC (50µM) as substrate. Correlations were determined by linear regression and expressed by Spearman coefficient (R_s) and levels of significance (p).

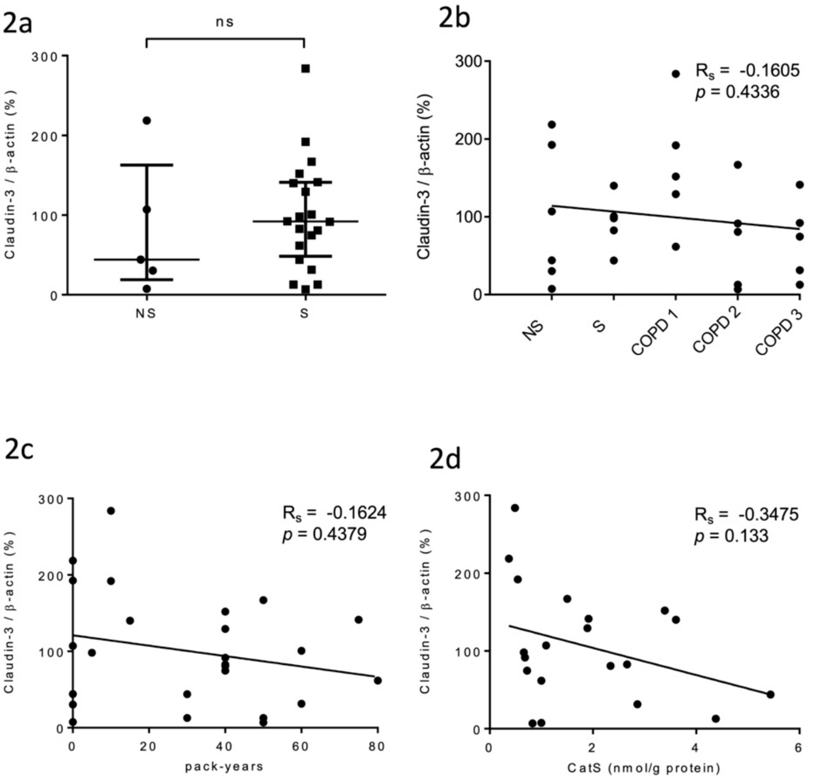

Supplementary Figure S2: Claudin-3 expression in lung specimen

a: The expression level of immunoreactive claudin-3 was analyzed by Western blot. Densitometric analysis of claudin-3 was performed (ImageJ software) using β-actin (load control) for normalization. Bars represent median ± quartile. No statistical significance (ns) was found (Mann–Whitney test).

b: Correlation between the expression level of claudin-3 and patient status.

c: Correlation between the expression level of claudin-3 and smoking history (packs/year). Correlations were determined by linear regression and expressed by Spearman coefficient (R_s) and levels of significance (p).

d: Correlation between CatS activity and the expression level of claudin-3. CatS concentration (tissue lysate, 10µg of total protein) was determined with E-64, using Z-Leu-Arg-AMC (50µM) as substrate. Correlations were determined by linear regression and expressed by Spearman coefficient (R_s) and levels of significance (p).

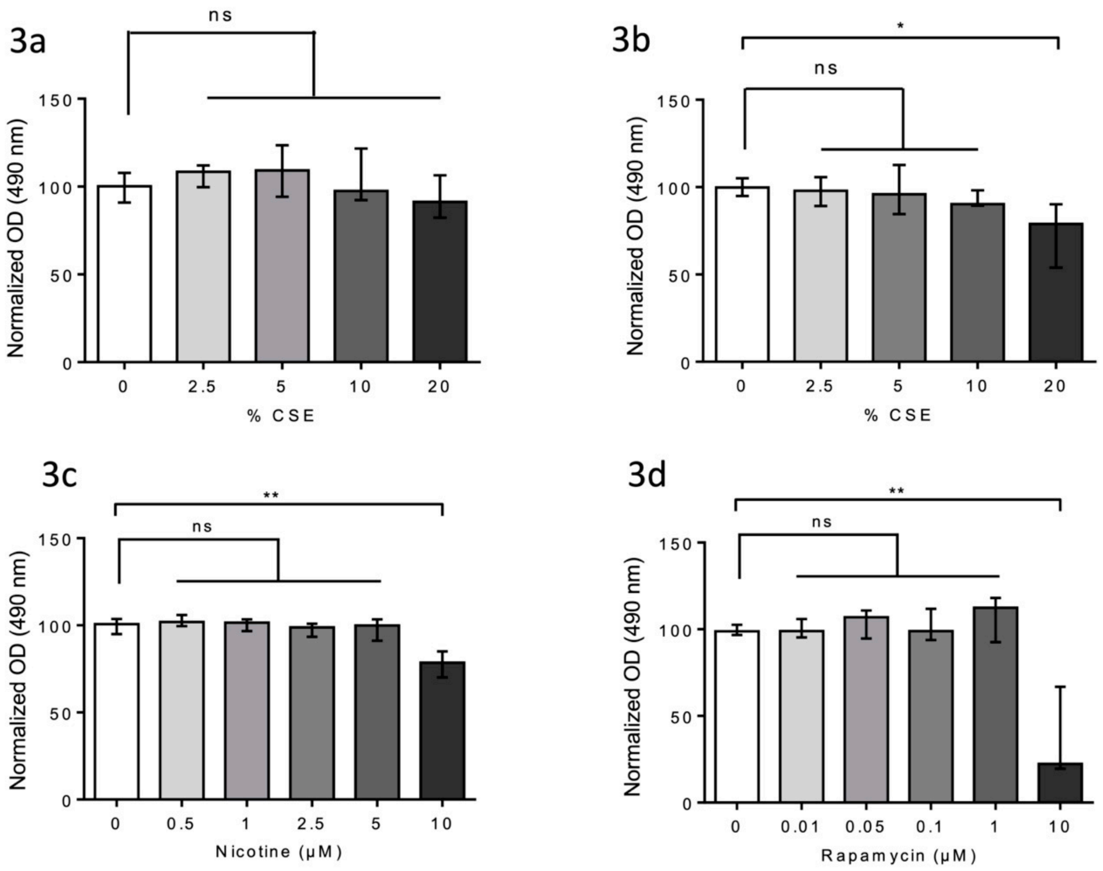

Supplementary Figure S3: Analysis of the cytotoxic effects of cigarette smoke extract (CSE), nicotine, and rapamycin on cell viability

a: 16HBE cells were treated with CSE (0-20%) for 12 h. Cell viability was further determined by an MTS assay (Promega, Madison, WI, USA).

b: THP1 cells were treated with CSE (0-20%) for 12 h before analysis of the cell viability (MTS assay).

c: THP1 cells were treated with nicotine (0-10 μM) for 12 h before analysis of the cell viability (MTS assay).

d: THP1 cells were treated with rapamycin (0-10 μM) for 12 h before analysis of the cell viability (MTS assay).

Bars represent median ± quartile. Results were normalized, using untreated cells as control. All the experiments were performed as three independent experiments, with each performed in triplicate.

Statistical significance was assessed using the non-parametric Kruskal–Wallis test (*, $p < 0.05$; **, $p < 0.01$; ns, non-significant.).

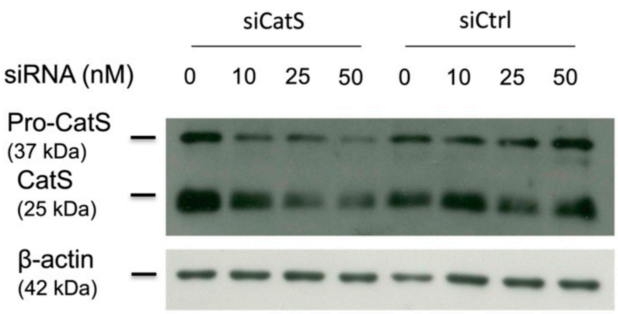

Supplementary Figure S4: Western blot analysis of the transient knockdown of cathepsin S by RNA interference.
Gene silencing of CatS was achieved by transfection of THP-1 cells by specific siRNAs in basal medium using HiPerFect transfection reagent (Qiagen SA) for 6h. Specific, predesigned small interfering RNAs for CatS (siCatS) and scrambled siRNA were as follows:
Hs_CTSS_1, AAGGATATATTCGGATGGCAA;
Hs_CTSS_2, AGGAATCTAATTATATCGAAA;
Hs_CTSS_3, CTGCCACATGTTCAAAGTACA;
Hs_CTSS_5, CTCATACGATCTGGGCATGAA.
Inhibition of transcription was optimal at 48 h, and the transient knockdown (circa 70%) of CatS was validated by WB (normalization: Human β-actin).

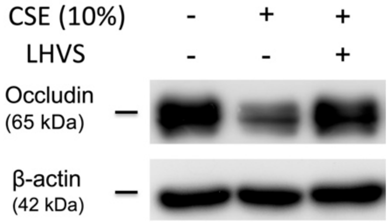

Supplementary Figure S5: Decrease in tight junction occludin following CSE-driven secretion of cathepsin S

THP-1 monocytes were seeded onto 12-well plates and differentiated into macrophages by adding 162 nM of PMA at day 0 [6]. In parallel, $7.5.10^4$ /cm² 16HBE cells were seeded on Transwell inserts (day 3). At day 5, inserts containing the 16HBE cells were placed in the upper culture chamber above 12-well plates seeded by THP-1 macrophages, while FCS was removed from the medium. At day 6, which corresponds both to the differentiation peak of THP-1 macrophages and to the confluence of epithelial cells, 10% CSE or 10% ethanol (control) was added to the FCS-free culture medium for 12 h. The same experiment was repeated in the presence of LHVS (100 nM). Experiments were performed in triplicate. Immunoblotting was performed as reported in the “Materials and Methods” section. Results were normalized to β-actin signal.
